# Ultrastructural characterization of the death process of hepatocytes in neonatal mouse liver

**DOI:** 10.1186/1476-5926-2-S1-S39

**Published:** 2004-01-14

**Authors:** Kazunobu Sasaki, Yuji Sonoda, Ichiro Kumano, Masumi Suda

**Affiliations:** 1 Department of Anatomy, Kawasaki Medical School, Matsushima 577, Kurashiki, 701-0192, Japan

## Introduction

Hematopoiesis in mouse fetal liver starts at 10 days of gestation and begins to decline after 15 days of gestation [1]. During postnatal hematopoietic involution, hepatocyte volume rapidly increases, and four types of specialized junctions; i.e., adherens junctions, desmosomes, tight junctions and gap junctions, appear to be fully developed in liver cells [2]. Neonatal livers examined by the TUNEL method contain numerous positive cells. Fetal liver contains cells undergoing apoptosis [3], and the majority of dying cells are hematopoietic cells. However, in addition, a few TUNEL-positive hepatocytes are observed in neonatal livers. The aim of this ultrastructural study was to clarify the hepatocyte death processes in mouse neonates.

## Methods

ICR mice from 13 days of gestation to 10 days after birth were used. For the TUNEL technique, paraformaldehyde-fixed paraffin-embedded liver sections were employed and examined under light microscopy. Cells undergoing DNA fragmentation have identified, using terminal deoxynucleotidyl transferase-mediated dUTP-biotin nick end labeling (TUNEL).

For ultrastructural examination, livers were fixed in Karnovsky's fixative and postfixed in osmium tetroxide, and toluidine blue stained semithin Epon sections were studied by light microscopy. After examination and photography, the sections selected for electron microscopy are re-embedded. Ultrathin sections were cut and mounted on formvar film-coated single hole copper grids for electron microscopic observation.

## Results

**Figure 1:**
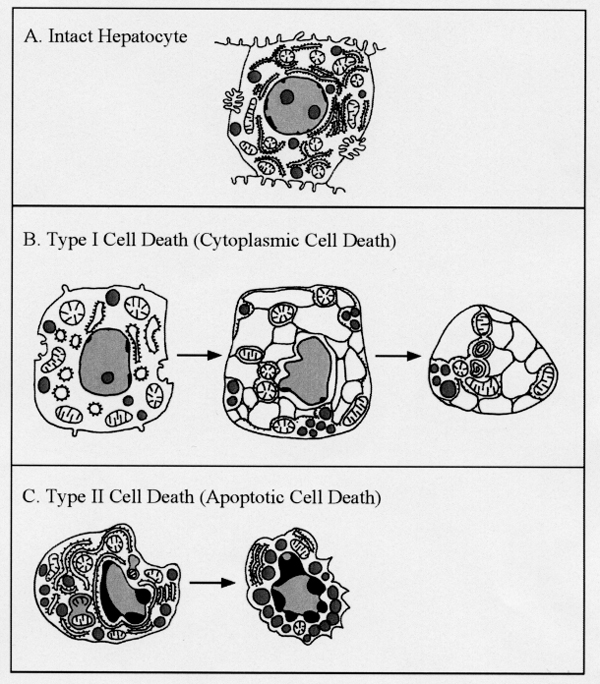
Ultrastructural features of (A) intact hepatocyte, (B) hepatocytes in the process of type I cell death, and (C) hepatocytes undergoing type II cell death in neonatal livers.

Analyses of section profiles of the hematopoietic compartments of fetal and neonatal livers revealed that liver hematopoiesis reached a peak at 13 days of gestation and thereafter gradually declined. After 15 days of gestation, hepatocyte volume rapidly increased, and the involuted hematopoietic foci were forced to move from inter-hepatocytic spaces to perisinusoidal space at the end of intrauterine life. TUNEL-positive hepatocytes could be observed after birth, specifically, between two and four days of age. As for ultrastructure, there were two different processes of cell death; type I and II (Fig. 1).

The early features of type I cell death appeared in the cytoplasm, which was characterized by dilated rough endoplasmic reticulum (RER) and distended perinuclear cisternae. Parallel cisternae of the RER were markedly expanded, fusing to produce large vacuolar profiles which entirely occupied the cytoplasm. The cytoplasm finally had a foamy appearance (Fig. 1-B). A progressive shrinkage in cell size and a parallel decrease in the size of the nucleus occurred. Then the nucleus might be totally dissolved within the cytoplasm. Foamy cytoplasmic bodies, consisting of round endoplasmic reticulum (ER) profiles, lost their junctional attachments and were occasionally observed either among hepatocytes or in perisinusoidal space apart from intact hepatocytes.

The early features of type II cell death, on the other hand, mainly appeared in the nucleus. Type II was characterized by compaction and margination of heterochromatin resulting in formation of sharply circumscribed masses and condensation of the cytoplasm (Fig. 1-C). Debris from dying cells was phagocytosed by hepatocytes as well as by sinusoidal macrophages. Cell debris showing a foamy appearance, which originated from type I cell death, frequently appeared in neonatal livers, but there were very few hepatocytes undergoing type II cell death.

## Discussion

Neonatal livers are characterized by the presence of two different morphological types of developmental cell death. Type I corresponds to cytoplasmic type degeneration and non-apoptotic death and type II to nuclear type cell death or apoptotic death.

On the basis of morphological studies on various organs from embryonic and fetal animals, several types of developmental cell death have been proposed [4,5]. In addition to the most well-known classical apoptosis, which is characterized by early nuclear collapse and massive condensation of chromatin [6], there is non-apoptotic cell death, or late nuclear damage type, which involves massive vacuolization of the cytoplasm with delayed collapse of the nucleus, with the cytoplasm being consumed by expansion of the lysosomal system [4,5]. Our results showed that most of the developmental cell death found in neonatal mouse livers is also characterized by massive vacuolization. However, this vacuolization is not due to expansion of lysosomal system but to dilatation of endoplasmic reticulum. Such dramatic ER dilation, followed by DNA fragmentation, could be provoked by apoptotic agents perturbing ER functions [7]. Since there was a much higher incidence of type I death than type II in neonatal livers, cytoplasmic type cell death due to inhibition of ER function may play a significant role not only in the formation of liver cell plates but also in the establishment of hepatic acinus.
